# PCK1 activates oncogenic autophagy via down-regulation Serine phosphorylation of UBAP2L and antagonizes colorectal cancer growth

**DOI:** 10.1186/s12935-023-02894-x

**Published:** 2023-04-16

**Authors:** Xiangyan Zhang, Geru Tao, Jie Jiang, Tingting Qu, Shuchao Zhao, Ping Xu, Ya’nan Zhao, Xiaoming Xing, Shucun Qin

**Affiliations:** 1 Department of Pathophysiology, Basic Medicine College, Qingdao University, Qingdao, 266071 China; 2 The Second Affiliated Hospital of Shandong First Medical University, Tai’an, 271000 People’s Republic of China; 3 Shandong First Medical University and Shandong Academy of Medical Sciences, Taishan Institute for Hydrogen Biomedicine, Tai’an, 271000 People’s Republic of China; 4 Department of Pathology, The Affiliated Hospital of Qingdao University, Qingdao, 266000 People’s Republic of China; 5 Laixi People’s Hospital, Qingdao, 266000 People’s Republic of China

**Keywords:** Phosphoenolpyruvate carboxykinase 1 (PCK1), Ubiquitin-associated protein 2-like (UBAP2L), Serine phosphorylation, Autophagy, Colorectal cancer

## Abstract

Phosphoenolpyruvate carboxykinase 1 (PCK1) is the rate-limiting enzyme in gluconeogenesis. PCK1 is considered an anti-oncogene in several human cancers. In this study, we aimed to determine the functions of PCK1 in colorectal cancer (CRC). PCK1 expression in CRC tissues was tested by western blot and immunohistochemistry analyses and associations of PCK1 level with clinicopathological characteristics and disease survival evaluated. Further, we studied the effect of PCK1 on CRC cell proliferation and the underlying mechanisms. Our results show that PCK1 is expressed at significantly lower levels in CRC than in control tissues. High PCK1 expression was correlated with smaller tumor diameter and less bowel wall invasion (T stage). Overexpression and knockdown experiments demonstrated that PCK1 inhibits CRC cell growth both in vitro and in vivo. Mechanistically, PCK1 antagonizes CRC growth via inactivating UBAP2L phosphorylation at serine 454 and enhancing autophagy. Overall, our findings reveal a novel molecular mechanism involving PCK1 and autophagy, and highlight PCK1 as a promising candidate therapeutic target in CRC.

**Supplementary Information:**

The online version contains supplementary material available at 10.1186/s12935-023-02894-x.

## Introduction

Colorectal cancer (CRC) is among the most common cancers and caused almost 10% of cancer deaths in 2020 [1]. Outcomes and life quality of patients with CRC have significantly improved due to early screening and systematic anti-cancer treatment; however, many patients exhibit intrinsic or de novo resistance to these therapies [2]. Therefore, novel therapies for CRC are urgently needed. Treatment strategies based on metabolic alterations of tumor cells represent a promising choice for patients with CRC.

Phosphoenolpyruvate carboxykinase (PCK; also known as PEPCK) is the rate-limiting enzyme of gluconeogenesis, and catalyzes the conversion of oxaloacetate (OAA) to phosphoenolpyruvate (PEP) [3]. PCK functions are performed by two isoforms: the cytoplasmic isoform, PCK1 or PEPCK-C, and the mitochondrial isoform, PCK2 or PEPCK-M [4]. PCK1 deficiency dis-regulates gluconeogenesis and causes an inherited metabolic disorder [5]. Further, PCK1 is involved in metabolic changes in cancer cells and is considered a potential treatment target in hepatocellular carcinoma [4, 6, 7] and clear cell renal cell carcinoma (ccRCC) [8]. PCK1 is significantly down-regulated in hepatocellular carcinoma relative to normal liver tissue, and patients with lower PCK1 expression have poor prognosis [7]. Regarding the underlying mechanism, PCK1 deficiency can promote hepatoma cell proliferation by dis-regulating the AMPK/CDK/Rb/E2F pathway and accelerating transition of the hepatoma cell cycle from G1 to S phase [4]. Moreover, Xu et al. demonstrated that PCK1 phosphorylated on serine (Ser) 90 could suppress the binding of sterols to INSIG and block translocation of the SCAP–SREBP complex to the Golgi apparatus, leading to hepatocellular carcinoma proliferation [6]. In ccRCC, overexpression of PCK1 can suppress tumor progression via inhibition of lactate dehydrogenase A (LDHA), and patients with high PCK1 expression had better prognosis [8]. In colon cancer, Montal et al. demonstrated that PCK1 was strongly expressed, and that PCK1 overexpression promoted glucose and glutamine utilization, thereby supporting anabolic metabolism and tumor proliferation [9]. PCK1 can also stimulate CRC cell proliferation and metastatic liver colonization by enhancing nucleotide synthesis in low oxygen environments [10]. Interestingly, we found PCK1 mRNA levels were significantly lower in CRC than those in control tissues, according RNA sequencing. Although several researchers have reported that PCK1 has important roles in CRC proliferation and metastasis via metabolic regulation, the signaling pathways and interactions affected by PCK1 are unclear.

Autophagy is an intracellular response to stress, which can avoid cell damage and maintain cellular homeostasis via degradation of dysfunctional proteins and organelles; however, autophagy is a double-edged sword in cancer pathogenesis, since its activation promotes cancer cell death during tumor initiation, while at advanced disease stages, autophagy can contribute to cancer cell growth [11]. The process of autophagy can be divided into two sections: autophagosome formation via lysosomes and autolysosome degradation by lysosomal hydrolase [12]. The ubiquitin- proteasome system is an important regulatory system in mammals, which can involve the formation of autophagosomes and participate in autophagy [12]. The ubiquitin-associated protein 2-like (UBAP2L) gene, also referred to as NICE-4, is a member of the ubiquitin-associated (UBA) domain family that maps to human chromosome 1q21.3 [13]. Previous studies have demonstrated that UBAP2L is an oncogene involved in various cancers, including glioma [13], gastric cancer [14], and uterine cancer [15]. Moreover, UBAP2L gene knockdown inhibits CRC cell growth via promoting apoptosis [16]. Previous studies on the function of UBAP2L have mainly focused on the protein expression level; however, the role of UBAP2L phosphorylation has received little attention.

In this study, we demonstrate that PCK1 is expressed at low levels in CRC tissues, while its overexpression suppresses CRC cell growth, and silencing PCK1 promotes tumor proliferation. Further, we identified a novel phosphorylation site at Ser 454 of UBAP2L via iTRAQ after overexpression of PCK1 in CRC cell lines. Our data demonstrate that PCK1 activates oncogenic autophagy via down-regulation of UBAP2L phosphorylation at Ser 454 and thereby suppresses CRC growth.

## Materials and methods

### Tissues samples and clinical data

Patients diagnosed with primary CRC (n = 491) who underwent surgical resection from 2014 to 2016 at the affiliated Hospital of Qingdao University were recruited to this study. Paraffin-embedded CRC specimens and matched adjacent non-tumor tissues were selected for analysis. Patients who had been treated with preoperative radiotherapy, chemotherapy, and/or targeted therapy were excluded. Clinicopathological data were collected as previously described [17]. Patients were followed up until December 2022, and the data recorded for analysis of patient prognosis. Four pairs of CRC and non-tumor specimens were collected for western blot (WB) analysis. The protocol of this study was approved by the Ethics Committee of the Affiliated Hospital of Qingdao University (QDFY-20130049).

### Cell lines and cell culture

Human CRC HCT116, RKO, SW480, Caco2, and LOVO cell lines were purchased from the Cell Bank of the Chinese Academy of Sciences (Shanghai, China), NCM460 were donated from the Central Laboratory of Qingdao University Affiliated Hospital. HCT116, RKO, SW480, Caco2 and NCM460 cells were cultured in Dulbecco’s modified Eagles’ medium (Gibco, C11995500BT, Thermo Fisher, USA) supplemented with 10%–20% fetal bovine serum (Gibco, 10,099-141c, Thermo Fisher, USA). LOVO cells were cultured in PRIM1640 (Gibco, C11875500BT, Thermo Fisher, USA). All cells were incubated in a humidified atmosphere at 37 °C with 5% CO_2_. The autophagy inhibitor, wortmannin (wort), Bafilomycin A1 (BAF), and the autophagy activator, rapamycin, were purchased from Med Chem Express.

### Plasmid construction and lentivirus transduction

A lentiviral vector containing the PCK1 sequence (PCK1-OE) was used for PCK1 overexpression, the lentiviral vector, pLV-shPCK1(PCK1-sh), was used for PCK1 knockdown; the control vector was lenti-Control (PCK1-Ctrl). Lentiviral vectors expressing the UBAP2L mutants were UBAP2L (Ser 445A/ 454A), (Ser 467A/477A), (Ser 445A), and (Ser 454A).

The transfection vectors, PCK1-OE, PCK1-Ctrl, PCK1-sh, UBAP2L Ser (445A/454 A), and Ser (467A/477A), were provided by Shanghai Gene Chem Corporation (Shanghai, China). The vectors, UBAP2L (Ser 445A), (Ser 454A), and GFP-RFP-LC3, were purchased from Hanheng BioChem Corporation (Shanghai, China).

Plasmids were transfected into RKO and SW480 cells, according to the manufacturer’s instructions. Transfection efficiency was tested by western blot (WB) assay. Cells transfected with lentiviruses were selected using puromycin (p8230, Solarbio, Beijing, China).

### WB analysis

Protein samples were extracted from cells and tissues as previously described [18]. Protein concentrations were determined using a BCA protein assay kit (Beyotime). Total proteins (20 μg) were separated by 10% or 12.5% SDS-PAGE. Then, proteins were transferred onto polyvinylidene fluoride membrane (Millipore, IPVH00010, MA, USA) at 100 V for 60 min. Membranes were blocked with 5% fat-free milk for 2 h at room temperature, then incubated at 4 °C overnight with primary antibodies against PCK1 (1:1000; Cat# 16754–1-AP; Proteintech, China), ATG5 (1:1000; Cat# 60061–1-lg; Proteintech, China), UBAP2L (1:1000; Cat# 40199; CST, USA), LC3B (1:1000; Cat# L7543, Sigma-Aldrich, USA), P62 (1:1000; Cat# 88588; CST, USA), p-UBAP2L (Ser454) (1:300; Cat# 3801-1 M; Qiangyao, Suzhou, China), and actin (rabbit polyclonal, 1:10000; Cat# E-AB-2058; Elabscience, China).

### iTRAQ and TMT analysis

iTRAQ was performed by Shanghai Applied Protein Technology, as previously described [19]. Briefly, peptides were digested using SDT buffer (4% SDS, 100 mM Tris–HCl, 1 mM DTT, pH7.6). The digested peptides were desalted on C^18^ Cartridges (Empore™ SPE Cartridges C^18^ (standard density), bed I.D. 7 mm, volume 3 ml, Sigma), concentrated by vacuum centrifugation, and reconstituted in 40 µl of 0.1% (v/v) formic acid. Then a filter-aided sample preparation (digestion) procedure was conducted, as follows: 200 μg of proteins for each sample were added into 30 μl SDT buffer (4% SDS, 100 mM DTT, 150 mM Tris–HCl, pH 8.0). Then, 100 μl iodoacetamide (100 mM in UA buffer) was added to reduce cysteine residues, and the samples were incubated for 30 min in darkness. Next, protein suspensions were digested with 4 μg trypsin (Promega) in 40 μl 25 mM NH_4_CO_3_ buffer overnight at 37 °C, and the resulting peptides collected. Proteins were separated by 12.5% SDS-PAGE and stained using Coomassie blue.

### LC–MS/MS analysis

Peptides were loaded onto a reverse phase trap column (Thermo Scientific Acclaim PepMap100, nanoViper C^18^) connected to a C^18^-reversed phase analytical column (Thermo Scientific Easy Column, 10 cm long, 75 μm inner diameter, 3 μm resin) in 0.1% formic acid buffer and separated with a linear gradient buffer (84% acetonitrile and 0.1% Formic acid) at a flow rate of 300 nl/min, controlled by IntelliFlow technology. After chromatographic separation, LC-MS/MS analysis was performed on a QExactive mass spectrometer (Thermo Scientific) coupled with Easy nLC (Proxeon Biosystems, Thermo Fisher Scientific) for 90 min.

### Cell proliferation assay

Cells were seeded into 96-well plates at a density of 1 × 10^3^ per well. Cell proliferation was assessed using a cell counting kit-8 (CCK-8) assay (Dojindo; Dalian, China) at the indicated time points, according to the manufacturer’s instructions. Light absorbance at 450 nm was measured using automated microplate reader (Infinite f200, Tecan, Australia) after incubation with 10 μl CCK-8 regent for 1.5 h at 37 °C.

### Colony formation assay

RKO and SW480 cells were seeded in 6-well plates at 7 × 10^2^ cells per well. Culture medium was replaced every 3 days. After culturing for 14 days, cells were washed and fixed with 4% paraformaldehyde for 30 min, stained with 0.1% crystal violet for 1 min, and counted.

### Electron microscopy

Cells were fixed with 2.5% glutaraldehyde (Servicebio Technology Co., Ltd, Wuhan, China) for 15 min at room temperature, and centrifuged at 2000 × g for 2 min. Then, cells were suspended in 2.5% glutaraldehyde for 1 h at room temperature. After dehydration, cells were embedded in epoxy resin. Ultrathin (90 nm) sections were cut and placed onto copper grids. Sections were stained with 1% uranyl acetate and 0.2% lead citrate, and examined using a JEOL-1200EX transmission electron microscope (Beijing, China).

### Immunofluorescence

Cells were transfected with GFP-RFP-LC3 and PCK1-OE, PCK1-sh, or PCK1-Ctrl plasmids, then inoculated onto a laser confocal Petri dish (15 mm, NEST) and observed using a confocal laser-scanning microscope (Leica, Wetzlar, Germany) at the indicated time points. Numbers of GFP-LC3 dots were counted in at least five independent visual fields.

### Immunohistochemistry (IHC)

IHC was performed to analyze paraffin-embedded CRC and adjacent tissue samples (n = 491). After deparaffinization and rehydration, sections were treated with Tris–EDTA (pH = 9.0) at 100 °C for 10 min. Sections were treated with 3% hydrogen peroxidase (10 min, room temperature), followed by incubation with primary antibody targeting PCK1 (1:600; Cat# 16754–1-AP; Proteintech, China), UBAP2L (1:600; Cat#40199; CST, USA), ATG5 (1:600; Cat# 60061–1-lg; Proteintech, China), P62 (1:600; Cat# 88588S; CST, USA), or UBAP2L (Ser 454) (1:300; Cat#3801-1 M; Qiangyao, Suzhou, China), for 1 h at 37 °C. Sections were then incubated with HRP-conjugated secondary antibody for 30 min, and a 3,3-diaminobenzidine (DAB) kit (ZSGB-Bio, ZL1-9017, Beijing, China) for 1 min. Finally, sections were counterstained with hematoxylin.

Scoring of IHC staining was based on the extent of positive tumor cells and the intensity of staining (0 = negative, 1 = weak, 2 = medium, or 3 = strong). The percentage of positive tumor cells was scored as 0 (negative), 1 (1 − 25%), 2 (26–50%), 3 (51–75%), or 4 (> 75%). The two scores were multiplied, and the resulting immune-reactive score (values from 0 to 12) used to classify samples into two categories: high (score, 5–12) and low (score, 1–4) expression level [10].

### Animal experiments

A murine xenograft model was used to investigate the roles of PCK1 and UBAP2L (Ser 454A) in tumor growth in vivo. Four-week-old BALB/c nude mice were purchased from Charles River (Beijing, China). Infected SW480 cells (4 × 10^6^) were injected into the shoulders of the nude mice. Mice were euthanized by cervical dislocation after inhalational CO_2_ when the maximum diameter of any tumor approached 1.5 cm. Tumor volume (V) was calculated by measuring the length (L) and width (W), following the equation: V = (L × W^2^) × 0.5. Animal experiment protocols were approved by the Shandong First Medical University (W202107060302).

### Statistical analysis

All data were evaluated using SPSS 19.0.0 software (SPSS, Chicago, IL, USA) and are expressed as mean ± SD. All experiments were repeated at least three times. Chi-square (χ2) or Fisher exact tests were used to evaluate the relationships between clinical pathological features and PCK1 expression. Analyses of disease-free survival (DFS) and overall survival (OS) were conducted using the Kaplan-Meier method with log-rank test. Multivariable analysis was performed using a Cox proportional hazard model. P < 0.05 was considered statistically significant.

## Results

### PCK1 expression and prognostic value in primary CRC

**Fig. 1:**
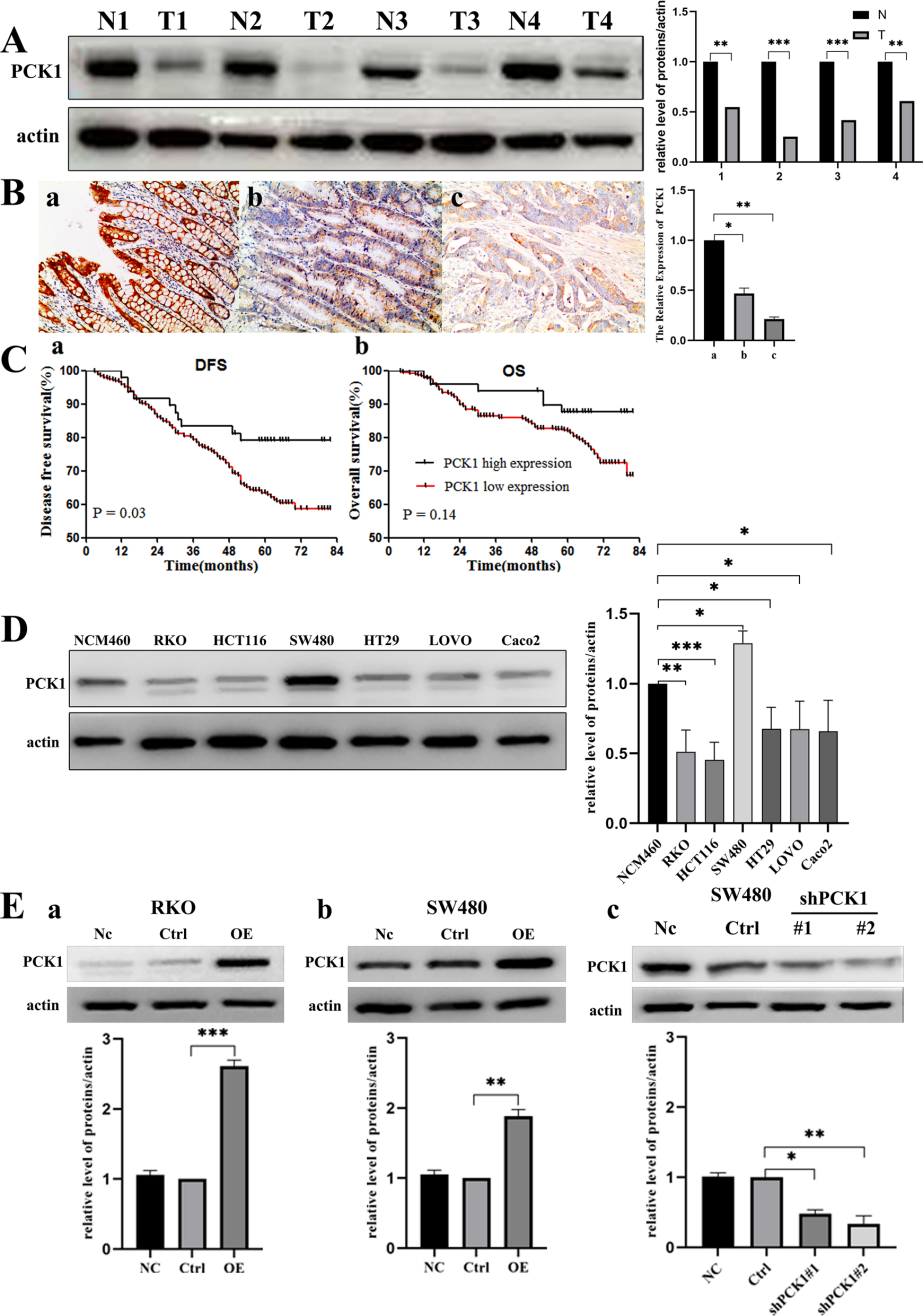
PCK1 expression and prognostic value in primary CRC. **A** PCK1 expression in fresh CRC tissues tested by western blot analysis. **B** Immunohistochemical staining for PCK1 expression in paraffin-embedded CRC and normal epithelium and adenoma tissues. Staining intensity was scored as strong in normal epithelial tissues (3 + , a), moderate in adenoma tissues (2 + , b), and negative in CRC tissues (1 + , c). All images are at 100 × magnification. **C** Survival curves for disease free survival (DFS) and overall survival (OS) in patients with stage I–III CRC, according to PCK1 expression. (a) DFS according to PCK1 expression; (b) OS according to PCK1 expression. **D** Western blot analysis of PCK1 protein expression in NCM460, HCT116, RKO, SW480, Caco2, and LOVO cells. **E** Western blot analysis was used to confirm transfection efficiency (a and b). (c) PCK1-shRNA (1#, and 2#) knockdown efficiency in SW480 cells. Actin was used as the control. *P < 0.05, **P < 0.01, and ***P < 0.001

PCK1 expression was examined by WB in 4 pairs of fresh CRC and normal epithelium tissue samples. As shown in Fig. 1A, PCK1 was expressed at significantly lower levels in CRC than in non-tumor specimens. Next, we tested the expression levels of PCK1 in 491 pairs of CRC and corresponding normal epithelium tissues, as well as 4 adenoma tissue samples, using an IHC assay. As shown in Fig. 1B, PCK1 was expressed at significantly lower levels in CRC than in normal epithelial tissues. Of CRC tissue samples, 446 exhibited low PCK1 expression compared with that in adjacent normal tissues (90.8%, 446/491 vs. 10.8%, 53/491, P < 0.001). According to Kaplan-Meier and log-rank test survival analysis, we found that factors significantly associated with DFS were tumor diameter (P = 0.011), tumor stage (P = 0.001), lymph node metastasis (P = 0.001), and PCK1 expression (P = 0.03) (Table1). Therefore, tumor diameter, tumor stage, lymph node metastasis, PCK1 expression, and tumor differentiation were entered into a multivariate prognostic Cox regression model. The results showed that low PCK1 expression was an independent factor predicting worse DFS, and tumors with low PCK1 expression were associated with a 2.23-fold increase in the risk of cancer recurrence or metastasis (hazard ratio = 2.23, 95% confidence interval [1.02–4.89], P = 0.033) (Table 2). PCK1 was not significantly associated with OS in univariable analysis (Fig. 1C).

**Table 1:** Univariate analysis of prognostic factors influencing disease free survival (DFS) in stage I–III CRC

	Disease-free survival			
Characteristics		HR	95%CI	P
Gender	Female vs Male	1.23	0.90–1.69	0.185
Age (year)	≤ 50 vs > 50	0.81	0.52–1.27	0.36
Location	Right side colon vs Left side colon vs Rectum			0.15
Mucin production	With vs Without	1.18	0.75–1.85	0.47
Tumor differentiation	Poor vs Moderate/well	1.46	1.0–2.13	0.052
Tumor diameter	≤ 5 cm vs >5 cm	1.49	1.10–2.03	0.011
Tumor stage	I + II vs III	1.88	1.38–2.57	0.001
Bowel wall invasion (T)	T1 + T2 vs T3 + T4	1.04	0.71–1.55	0.84
Lymph node metastasis (N)	With vs Without	1.88	1.38–2.57	0.001
Lymphovascular invasion	YES vs No	1.25	0.89–1.75	0.201
Alcohol intake	Never vs Ever	0.76	0.52–1.09	0.14
Smoking	Never vs Ever	0.85	0.61–1.20	0.35
Cancer family history	Yes vs No	1.29	0.83–2.00	0.26
Colorectal family history	Yes vs No	1.19	0.64–2.21	0.59
MSI status	MSI vs MSS	1.02	0.65–1.59	0.94
KRAS status	Mutation vs Wildtype	1.12	0.83–1.53	0.46
PCK1	Low vs High	1.71	1.05–2.76	0.03

**Table 2:** Independent prognostic factors correlating with disease free survival (DFS) in stage I–III CRC by Cox’s regression analysis

Characteristics	HR (95% CI)	P
PCK1	2.23 (1.02–4.89)	0.033
Tumor diameter	2.15 (1.34–3.44)	0.001
Tumor stage	1.91 (1.16–3.14)	0.022
Lymph node metastasis	1.18 (0.82–1.68)	0.378

### PCK1 status and associations with clinicopathological characteristics

Associations between PCK1 status and clinicopathological characteristics are shown in Table 3. PCK1 expression was significantly associated with smaller tumor diameter (tumor diameter ≤ vs. > 5 cm; 14.13% vs. 6.76%, P = 0.009), less bowel wall invasion (T stage) (T1 + T2 vs. T3 + T4; 23.07% vs. 8.00%, P = 0.002), and history of alcohol intake (never vs. ever; 12.40% vs. 4.81%, P = 0.031) (Table 3).

**Table 3:** Correlations between PCK1 expression and clinicopathological characteristics in patients with CRC (n = 491)

PCK1 expression				
Characteristics	Number	High (%)	Low (%)	P
Gender				
Male	299	35 (11.71%)	264 (88.29%)	0.26
Female	192	18 (9.38%)	174 (90.63%)	
Age (year)				
≤ 50	64	8 (12.5%)	56 (87.5%)	0.67
> 50	427	45 (10.54%)	382 (89.46%)	
Location				
Right side colon	112	15 (13.39%)	97 (86.61%)	0.53
Left side colon	93	8 (8.6%)	85 (91.4%)	
Rectum	286	30 (10.49%)	256 (89.51%)	
Mucin production				
With	46	6 (13.04%)	40 (86.96%)	0.48
Without	425	47 (11.06%)	378 (88.94%)	
Tumor differentiation				
Poor	106	9 (8.49%)	97 (91.51%)	0.25
Moderate/well	385	44 (11.43%)	341 (88.57%)	
Tumor diameter				
≤ 5 cm	269	38 (14.13%)	231 (85.87%)	0.009
>5 cm	222	15 (6.76%)	207 (93.24%)	
Tumor stage				
I + II	288	24 (8.33%)	264 (91.67%)	0.062
III	203	29 (14.29%)	174 (85.71%)	
Bowel wall invasion (T)				
T1 + T2	91	21 (23.07%)	70 (76.93%)	0.002
T3 + T4	400	32 (8.00%)	368 (92.00%)	
Lymph node metastasis (N)				
Without	288	24 (8.33%)	264 (91.67%)	0.062
With	203	29 (14.29%)	174 (85.71%)	
Lymphovascular invasion				
No	335	35 (10.45%)	300 (89.55%)	0.76
Yes	156	18 (11.54%)	138 (88.46%)	
Alcohol intake				
Never	387	48 (12.40%)	339 (87.60%)	0.031
Ever	104	5 (4.81%)	99 (95.19%)	
Smoking				
Ever	137	16 (11.68%)	121 (88.32%)	0.75
Never	354	37 (10.45%)	317 (89.55%)	
CRC family history				
Yes	9	2 (22.22%)	7 (77.78%)	0.28^*^
No	119	13 (10.92%)	106 (89.08%)	
Unknown	363			
MSI status				
MSI	68	4 (5.88%)	64 (94.12%)	0.21
MSS	423	49 (11.58%)	374 (88.42%)	
KRAS status				
Mutation	212	21 (9.91%)	191 (90.09%)	0.66
Wildtype	279	32 (11.47%)	247 (88.53%)	

### PCK1 inhibits CRC cell proliferation

**Fig. 2:**
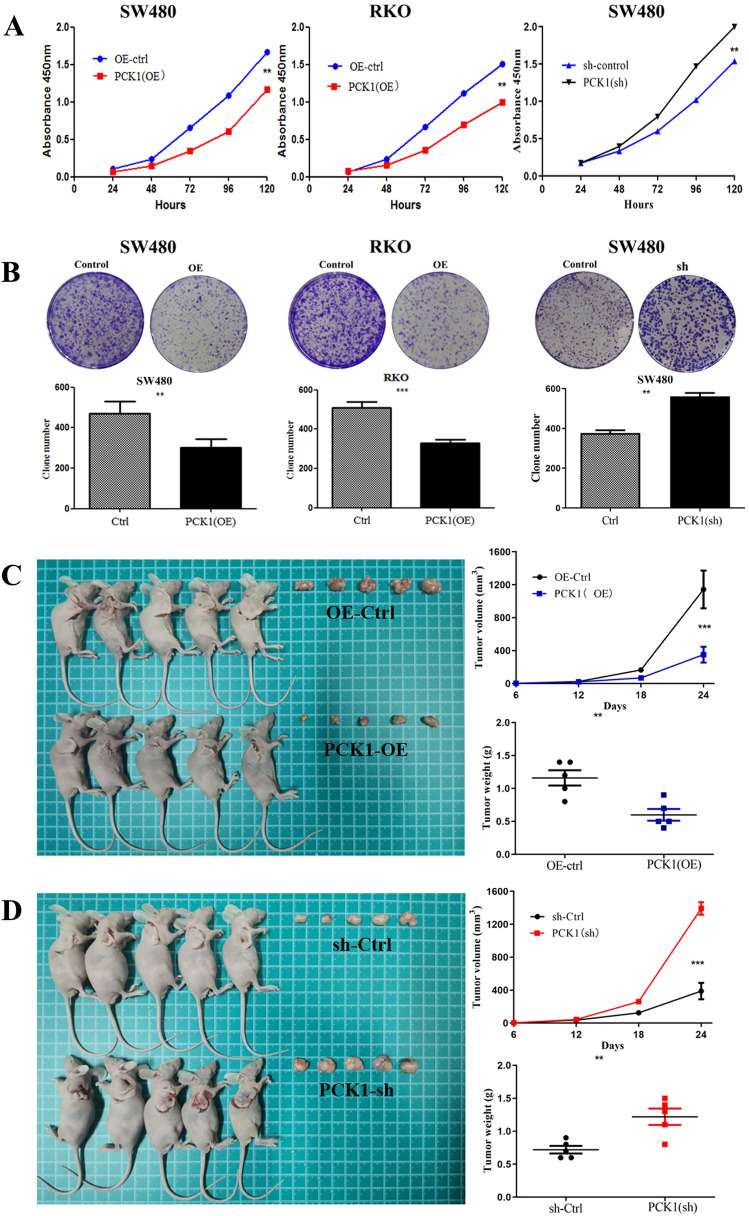
Over-expression of PCK1 inhibits CRC cell proliferation in vitro and in vivo. **A** Viability of SW480 and RKO cells in the Ctrl, PCK1-OE, and PCK1-sh groups over time (24, 48, 72, 96 and 120 h), measured by CCK8 assay (450 nm absorbance). **B** Colony formation of SW480 and RKO cell lines in the Ctrl, PCK1-OE, and PCK1-sh groups at 15 days. **C** Tumor volume and weight were measured 24 days after tumor cells were injected into BALB/c- nude mice. *P < 0.05, **P < 0.01, and ***P < 0.001

To assess the role of PCK1 in CRC proliferation, we first examined endogenous PCK1 levels in several CRC cell lines. We found low endogenous PCK1 expression in the RKO, HCT116, LOVO, CaCo2, and HT29 cell lines and high expression levels in SW480 cells (Fig. 1D). Next, the PCK1-OE and PCK1-Ctrl vectors were transfected into RKO and SW480 CRC cell lines and the knockdown vector (PCK1-sh) was transfected into SW480 cells, based on their endogenous PCK1 expression levels. As shown by WB, PCK1 expression was significantly up-regulated in PCK1-OE transfected cells and down-regulated in PCK1-sh transfected cells (Fig. 1E). Compared with PCK1-Ctrl cells, overexpression of PCK1 inhibited CRC cell growth, while PCK1 knockdown using PCK1-sh promoted CRC cell growth (Fig. 2A, P < 0.01). Further, colony formation assays showed that PCK1 overexpression decreased the colony number of RKO (PCK1-OE vs. Ctrl: 341 ± 19.1 vs. 492 ± 38.4, P < 0.01) and SW480 (PKC1-OE vs. Ctrl: 352 ± 23.9 vs. 548 ± 23.9, P < 0.001) cells, while PCK1 knockdown clearly increased cell colony number (PCK1-sh vs. Ctrl: 561 ± 16.9 vs. 377 ± 13.8, P < 0.01) (Fig. 2B).

Next, we explored the role of PCK1 in CRC cell growth using a xenograft model. SW480 cells transfected with different vectors (PCK1-OE and PCK1-sh) were injected into the shoulders of nude mice; cells transfected with PCK1-Ctrl vector were injected as a control. Tumors were formed approximately 6 days later, then collected and measured on day 24. As shown in Fig. 2C and D, tumors formed by PCK1-OE cells were smaller than those formed by control cells (245 ± 25.67 vs. 1270 ± 24.53 mm^3^, P = 0.001), while tumor volume (1367 ± 23.68 vs. 307 ± 31.39 mm^3^, P = 0.001) and weight (1.22 ± 0.12 vs. 0.72 ± 0.06 g, P = 0.006) were notably increased in the PCK1-sh group compared with those in controls. Overall, these data indicate that PCK1 overexpression could inhibit CRC cell growth both in vitro and in vivo.

### PCK1 antagonizes CRC growth by activating autophagy

**Fig. 3:**
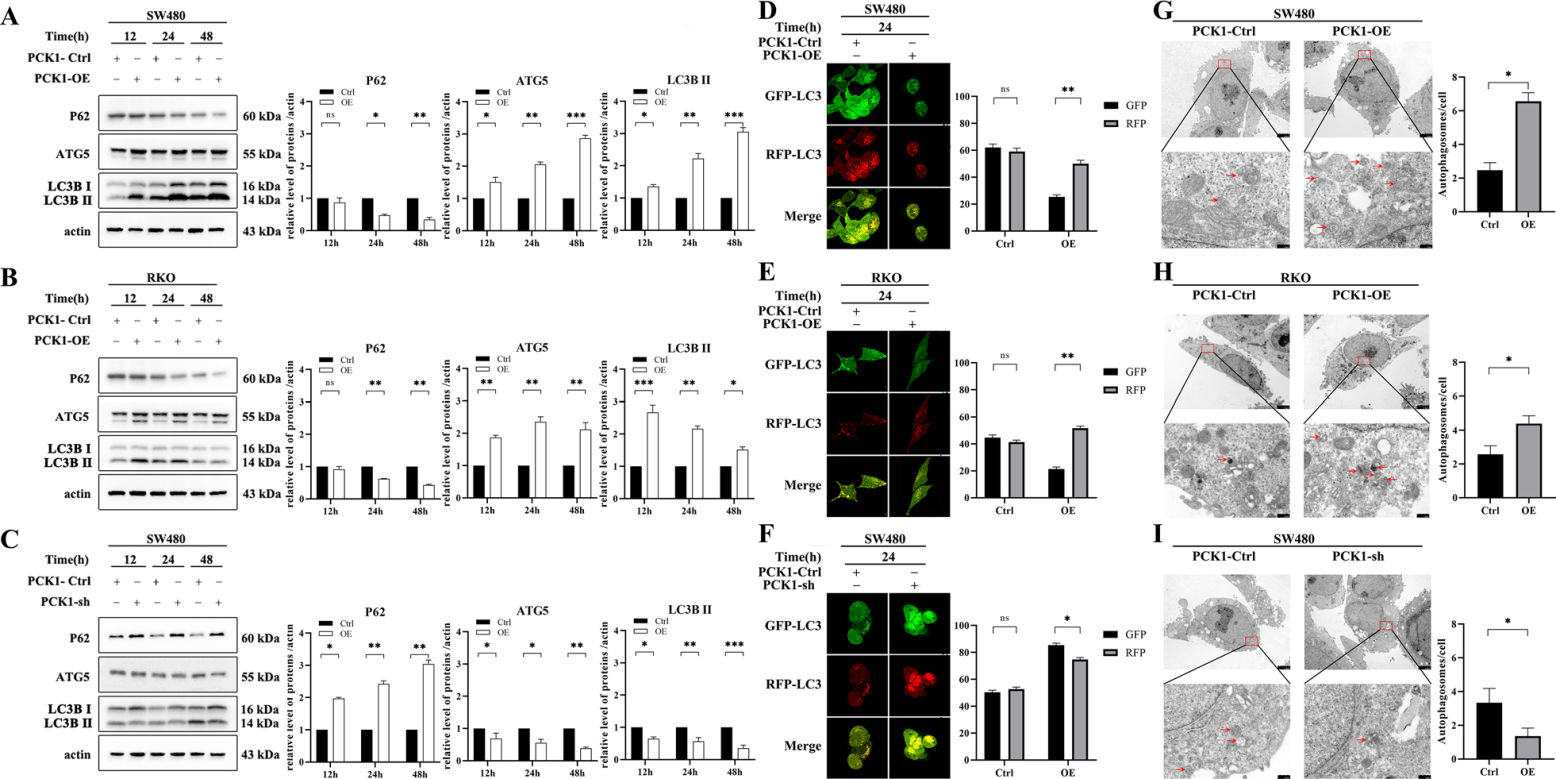
PCK1 affects colorectal cancer cell autophagy. **A** and **B** Western blot analysis of P62, ATG5, and LC3B protein expression in SW480 (**A**) and RKO (**B**) cells transfected with PCK1-OE or PCK1-Ctrl for 12, 24, and 48 h. **C** Western blot analysis of P62, ATG5, and LC3B protein expression in SW480 cells transfected with PCK1-sh or PCK1-Ctrl for 12, 24, and 48 h. Actin was used as a control. **D** and **E** Immunofluorescence dots in SW480 (**D**) and RKO (**E**) cells transfected with PCK1-OE or PCK1-Ctrl and GFP-RFP-LC3 for 24 h. **F** Immunofluorescence dots in SW480 cells transfected with PCK1-sh or PCK1-Ctrl and GFP-RFP-LC3 for 24 h. **G** and H) Electron microscope images showing autophagic vesicles in SW480 (**G**) and RKO (**H**) cells transfected with PCK1-OE or PCK1-Ctrl for 24 h. **I** Electron microscope images showing autophagic vesicles in SW480 cells transfected with PCK1-sh or PCK1-Ctrl for 24 h. *P < 0.05, **P < 0.01, and ***P < 0.001

Autophagy is a ubiquitous catabolic process that degrades damaged proteins and organelles via the autophagosome pathway [20], and is proven to contribute to carcinogenesis of several cancers. LC3-I turnover to LC3-II is a marker of an autophagy process; therefore, we detected the expression of autophagy-associated proteins, including LC3B, ATG5, and P62, to explore whether PCK1 may be involved in autophagy. LC3-II/LC3-I and ATG5 expression levels were significantly increased in cells overexpressing PCK1, while P62 protein expression was significantly reduced. Conversely, significant decreases in LC3-II/LC3-I and ATG5 levels were observed in cells with PCK1 knocked down, while P62 protein expression was increased (Fig. 3A–C). Since activation of autophagy by PCK1 overexpression was most pronounced 24 h after transfection of CRC cells, we selected the 24 h time point for all subsequent studies. Immunofluorescence analysis showed that the number of GFP-LC3 dots was significantly decreased in cells overexpressing PCK1 for 24 h, while the number of GFP-LC3 dots was significantly increased in PCK1-sh cells (Fig. 3D–F). Using electron microscopy, we also observed that the cell membrane fragment and autophagosomes increased in cells overexpressing PCK1 (Fig. 3G–I). These results provide evidence that PCK1 can induce CRC cell autophagy.

**Fig. 4:**
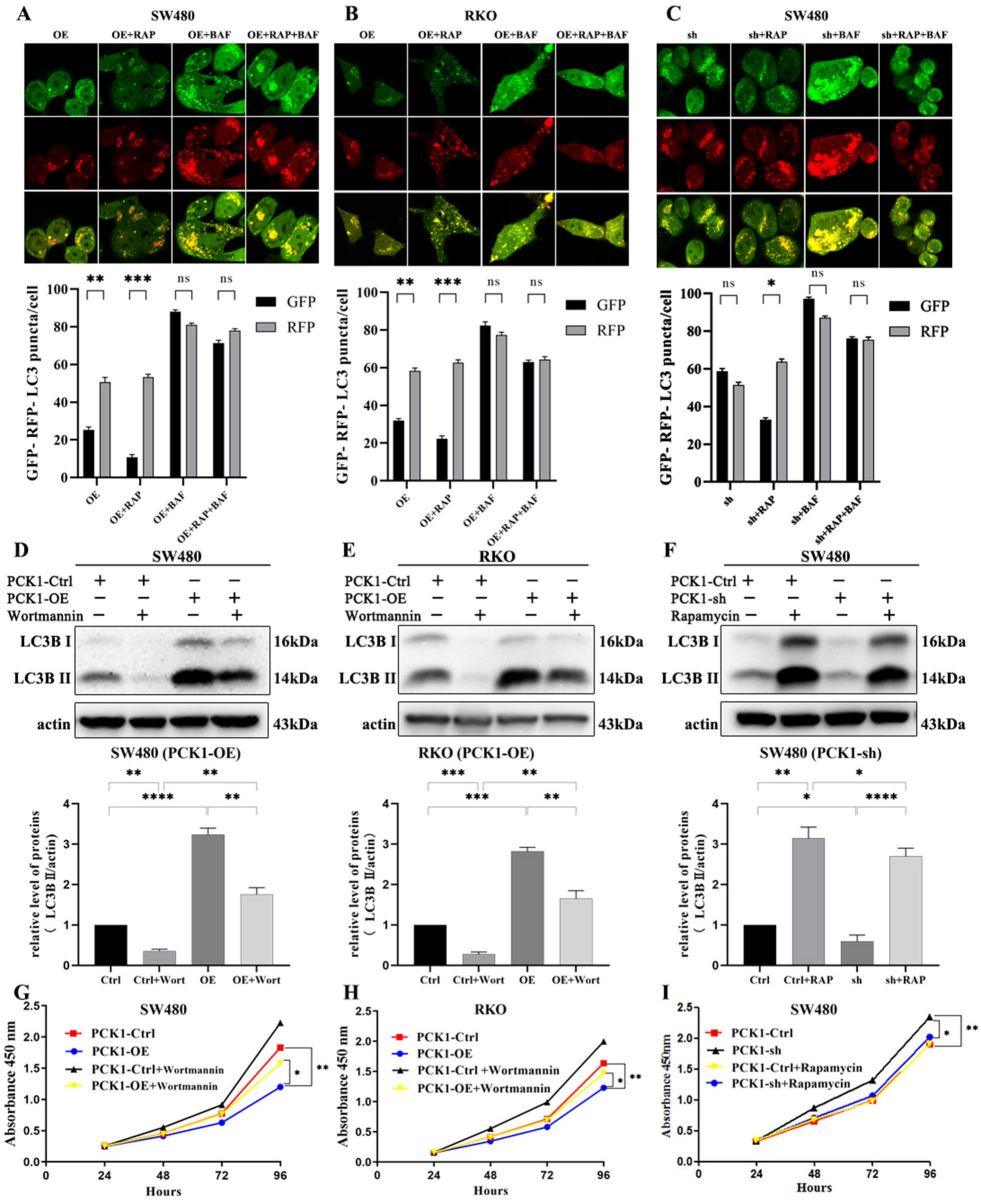
Effect of PCK1 expression level on CRC cell growth via regulating autophagy. **A** and **B** Autophagic flux of PCK1-OE SW480 (A) or PCK1-OE RKO (B) cells after rapamycin or BAF treatment or a combination of both treatments for 4 h. **C** Autophagic flux of PCK1-sh SW480 cells after rapamycin or BAF treatment or a combination of both treatments for 4 h. **D** and **E** LC3B protein expression after treatment of SW480-OE (D) or RKO-OE (E) cells with phosphate-buffered saline (control) or a PI3K inhibitor (wortmannin, 1 mM). **F** LC3B protein expression after treatment with phosphate-buffered saline (control) or rapamycin (2.5 mM) in SW480-sh cells. Actin was used as a control. **G** to **I** Comparison of cell viability determined by CCK8 assay. SW480 (**G**) and RKO (**H**) cells were transfected with PCK1-OE or PCK1-Ctrl and treated with phosphate-buffered saline (control) or wortmannin (1 mM) for 24 h. **I** SW480 cells were transfected with PCK1-sh or PCK1-Ctrl, and treated with phosphate-buffered saline (control) or rapamycin (2.5 mM) for 24 h. *P < 0.05, **P < 0.01, and ***P < 0.001

LC3B-II accumulation indicates increased autophagosome formation or a block of autophagy-lysosome fusion. To distinguish between these two different processes, LC3B-II levels were tested in the presence of rapamycin or BAF. We first tested LC3B-II expression at 0, 1, 3, and 6 h, and found that it clearly increased after BAF treatment for 1 h. Moreover, BAF significantly increased LC3B II expression in PCK1-OE cells relative to that in PCK1-Ctrl and PCK1-sh cells (Additional file 1: Fig. S1). Rapamycin also increased LC3B expression in CRC cells (Additional file 1: Fig. S2). Next, we evaluated the kinetics of autophagic flux after rapamycin or BAF treatment for 1 h and found that rapamycin significantly decreased the numbers of GFP-LC3 dots in Ctrl, PCK1-OE, and PCK1-sh cells, while BAF increased the numbers of GFP-LC3 dots in these cells (Fig. 4A–C and Additional file 1: Fig. S3). Collectively, these results indicate that PCK1 can increase autophagosome formation and activate autophagy, while silencing of PCK1 can block autophagic flow.

To clarify the effect of autophagy in PCK1-OE CRC cells, CCK8 assays were conducted after treatment of PCK1-OE CRC cells with wort (a PI3K inhibitor) from 24 h (Fig. 4D and E). The results showed that wort significantly reversed PCK1-OE suppression of CRC cells (Fig. 4G and H). We also treated PCK1-sh cells using rapamycin (Fig. 4F); as expected, the promotion of cell viability induced by PCK1-sh was reversed, as determined by CCK-8 assay (Fig. 4I). These results indicate that PCK1 suppresses CRC cells growth via activation of autophagy.

### PCK1 down-regulation Serine 454 phosphorylation of UBAP2L

**Fig. 5:**
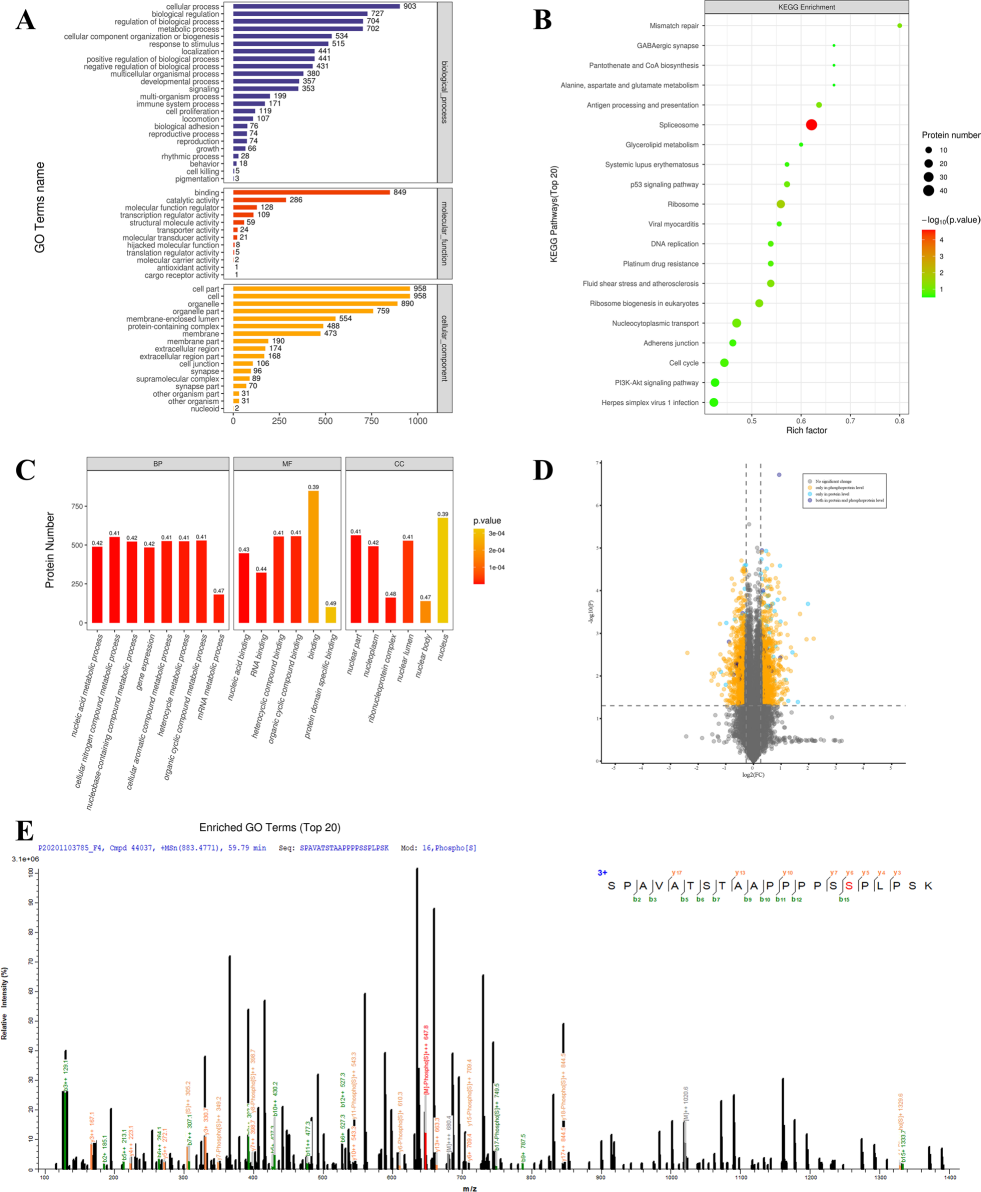
iTRAQ technical flow chart and localization of the identified proteins and phosphorylation events in RKO cells, according to PCK1-OE and PCK1-Ctrl. **A**–**D** GO analysis of the involved biological processes. **E** iTRAQ technical flow for assessing the effect of PCK1 over-expression on serine phosphorylation of UBAP2L at 445/454/467/477

To explore the mechanism underlying PCK1-induced autophagy, we analyzed chip-based iTRAQ protein and iTRAQ phosphoprotein arrays of RKO cells transfected with PCK1-OE and PCK1-Ctrl. LC–MS/MS analysis revealed a series of changes in multiple functional proteins, including molecules in the proteasome pathway, molecular function regulators, and trafficking proteins, among others (Fig. 5). Here, we focused on mechanisms involved in the autophagy-lysosome pathway. We identified the protein, UBAP2L, whose expression levels remained stable, but had decreased phosphorylation levels in PCK1-OE CRC cells; therefore, we analyzed the UBAP2L Ser 445, Ser 454, Ser 467, and Ser477 phosphorylation sites, according to the positions of the modifications in the peptide and phosphorylation site probability.

### Abrogation of Ser 454 phosphorylation of UBAP2L inhibits CRC cell proliferation

**Fig. 6:**
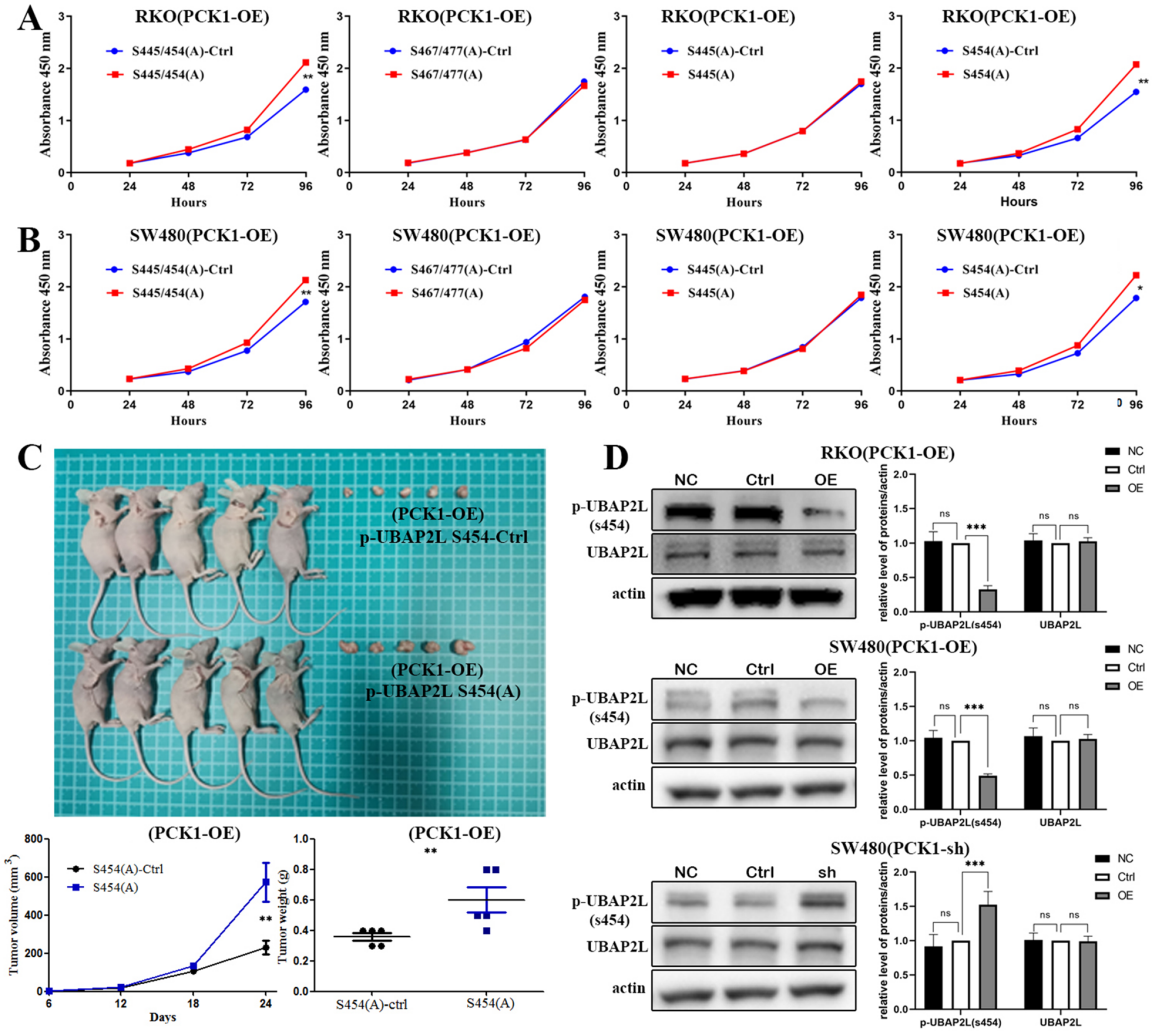
PCK1 over-expression inhibits CRC cell proliferation via decreasing Ser phosphorylation at UBAP2L 454. **A** and **B** CCK8 assays of PCK1-OE CRC cells with or without p-UBAP2L Ser 445, Ser 454, Ser 467, and Ser 477 mutations. **C** Calculated tumor volumes and weights in the p-UBAP2L Ser 454 ctrl and p-UBAP2L Ser 454 (**A**) groups 24 days after tumor cells were injected into the BALB/c- nude mice. (**D**) UBAP2L Ser 454 phosphorylation levels were analyzed by western blot analysis in PCK1-OE and PCK1-sh CRC cells. Actin was used as a control. *P < 0.05, **P < 0.01, and ***P < 0.001

**Fig. 7:**
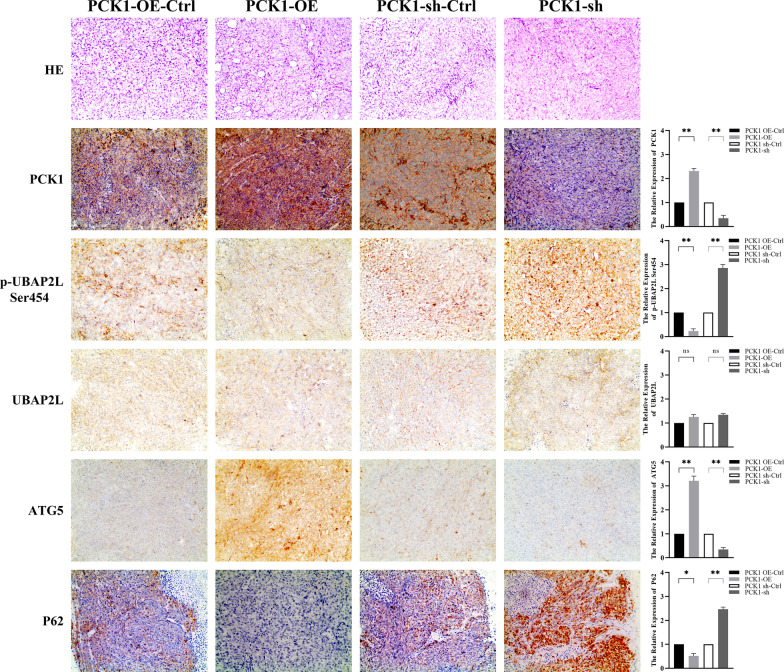
IHC analysis of PCK1, P62, ATG5, UBAP2L, and p-UBAP2L Ser454 expression in PCK1-OE and PCK1-KD tumor tissues. All images are at 100 × magnification. *P < 0.05, **P < 0.01, and ***P < 0.001

Whether UBAP2L phosphorylation is increased in CRC cells is unknown. Therefore, we conducted point mutation of UBAP2L phosphorylation sites to determine whether eliminating their phosphorylation would influence CRC cell proliferation. To understand if kinase activity to phosphorylate UBAP2L is necessary for CRC cell growth, we mutated the UBAP2L Ser 445/454 and Ser 467/477 sites in pairs and expressed mutated Ser 445/454 (A) and Ser 467/477 (A) UBAP2L at levels comparable with those of wild-type UBAP2L in PCK1-OE CRC cells. CCK8 assays showed that Ser 467/477 (A) mutation did not significantly influence cell proliferation relative to controls, while cell proliferation was significantly promoted on abrogation of Ser 445/454 phosphorylation by the Ser 445/454(A) mutations (Fig. 6A and B). This result indicates that UBAP2L phosphorylation is involved in CRC cell proliferation. Since Ser 445/454 phosphorylation is proven to promote CRC cell growth, we sought to clarify whether Ser 445 or Ser 454 phosphorylation was significant for this role by expressing Ser 445(A) and Ser 454(A) mutants separately in PCK1-OE CRC cells. CCK8 assays and animal experiments showed that Ser 454, rather than Ser 445, phosphorylation regulated CRC cell proliferation. Tumor volume (654 ± 24.3 vs. 169 ± 19.5 mm^3^, P = 0.001) and weight (0.6 ± 0.08 vs. 0.36 ± 0.02 g, P = 0.025) were notably increased in the p-UBAP2L at Ser454 (A) group compared with the p-UBAP2L at Ser454 ctrl group (Fig. 6C, P < 0.01). Therefore, abrogated phosphorylation of UBAP2L Ser 454 could reverse the inhibition caused by PCKI-OE in vitro and in vivo. To analyze UBAP2L Ser phosphorylation at the protein level, a specific antibody against S454-phosphorylated UBAP2L was developed. Using this antibody, we confirmed that expression of UBAP2L phosphorylated at S454 was significantly down-regulated in PCK1-OE CRC cells and significantly increased in PCK1-sh CRC cells (Fig. 6D). Tumors were also analyzed by IHC. IHC analysis showed PCK1 and ATG5 was up-regulated, while P62 and p-UBAP2L Ser454 expression was down- regulated in PCK1-OE tumor tissues (Fig. 7).

### PCK1-induced autophagy inhibits CRC cell growth via down-regulation of UBAP2L phosphorylation on Ser 454

**Fig. 8:**
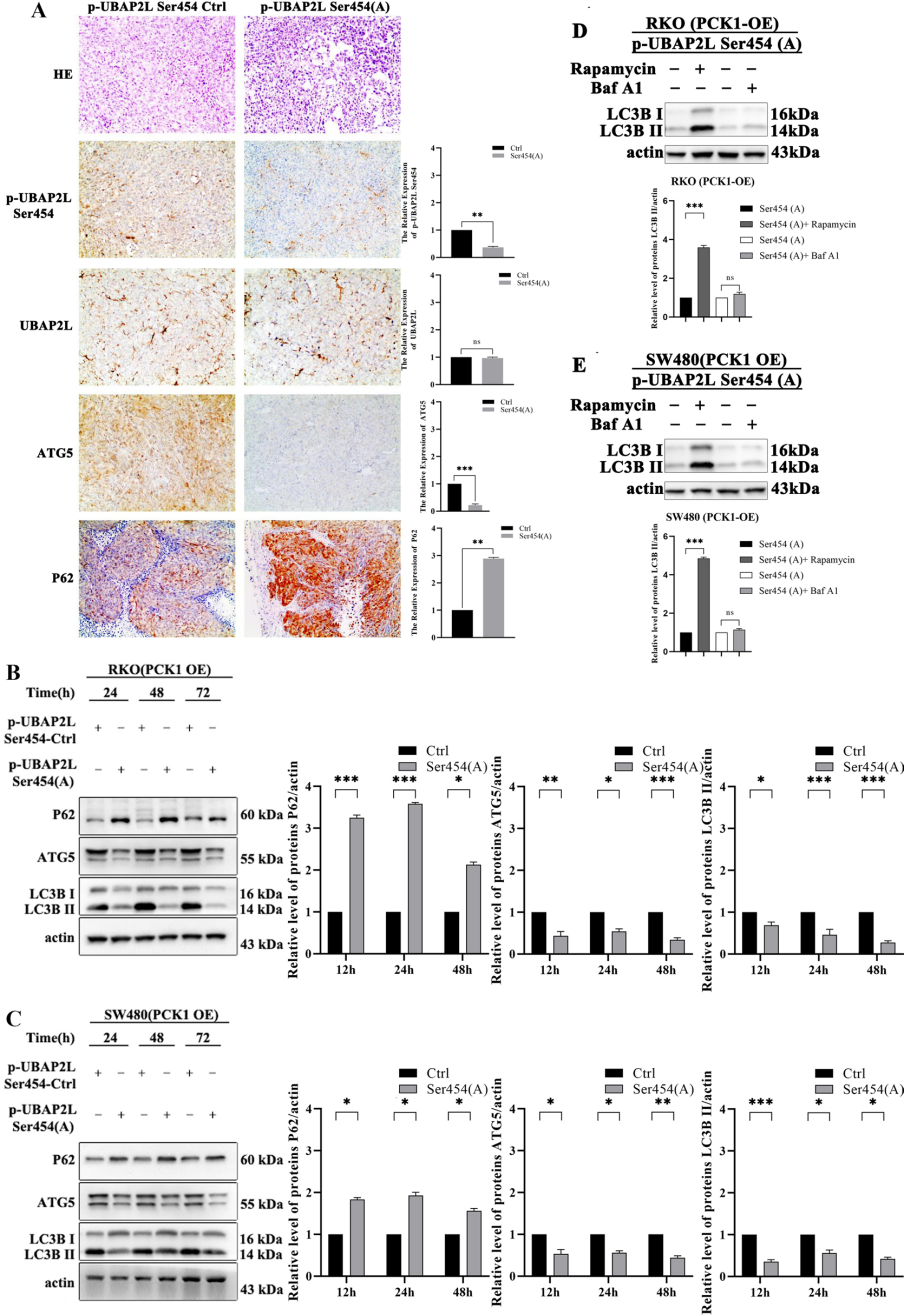
PCK1-induced autophagy inhibits CRC cell growth via down-regulation of UBAP2L Ser 454 phosphorylation. **A** IHC analysis p-UBAP2L Ser 454, UBAP2L, ATG5, and P62 expression in the p-UBAP2L Ser 454 (**A**) tumor and control groups. **B**–**C** P62, ATG5, and LC3B protein expression in PCK1-OE RKO (**B**) and PCK1-OE SW480 (**C**) cells transfected with Ser 454-Ctrl and Ser 454 (**A**) for 24, 48, and 72 h. **D** and **E** LC3B expression in Ser 454-Ctrl and Ser 454 (**A**) cells after treatment with rapamycin or BAF for 4 h. Actin was used as a control. *P < 0.05, **P < 0.01, and ***P < 0.001

As phosphorylation of UBAP2L on Ser 454 is involved in PCK1-induced growth inhibition, we explored whether abrogation of Ser 454 phosphorylation could partially reverse the autophagy effects induced by PCK1 overexpression. UBAP2L phosphorylation at Ser 454 was abrogated by infecting PCKI-OE with lentivirus expressing Ser 454(A). As expected, the expression levels of P62, ATG5, and LC3B-II were significantly reversed in PCKI-OE cells expressing UBAP2L with the Ser 454(A) mutation (Fig. 8A–C). To confirm the inhibitory effect of Ser 454, we tested LC3B levels in PCK1 OE cells (with or without p-UBAP2L mutation) after treatment with rapamycin or BAF. As expected, rapamycin increased the expression of LC3B, while LC3B did not accumulate significantly after treatment with BAF (Fig. 8D, E). Taken together, the above results suggest that PCK1-induced autophagy antagonizes CRC growth via down-regulation of UBAP2L Ser 454 phosphorylation.

## Discussion

Metabolic reprogramming is a hallmark of tumor cells, and interest in the metabolic changes associated with tumor proliferation and metastasis has been renewed in recent years [21, 22], and targeting metabolism for tumor treatment has attracted increasing attention. Evidence of the significance of PCK1 in tumor development and progression is accumulating. PCK1 can induce tumor cell proliferation and metastasis by disrupting cellular metabolism [4, 10, 23]. According to our previous study, RNA sequencing data demonstrated that PCK1 mRNA was clearly expressed at lower levels in CRC tissues than in control tissues. Therefore, we hypothesized that PCK1 might function as an anti-oncogene. Moreover, information on PCK1 status in Chinese patients with CRC was limited. Therefore, we explored the function of PCK1 in CRC oncogenesis, and evaluated the prognostic role of PCK1 in patients with primary CRC. Our data provide evidence that PCK1 overexpression can inhibit tumor cell growth via down-regulation of UBAP2L Ser 454 phosphorylation and increasing autophagy, and that low PCK1 expression is an independent predictive factor for CRC recurrence and metastasis.

Even under oxygen-sufficient conditions, tumor cells can use glycolysis to produce energy, a phenomenon known as the "Warburg effect". Anaerobic metabolism and glycolysis are significantly more active in malignant tumor cells than in normal cells [24, 25]. Zhang et al. showed that PCK1 is down-regulated by P53 in hepatocellular carcinoma and CRC cells, and that mutation of P53 directly activates expression of the NAD^+^-dependent histone deacetylase, sirtuin 6 (SIRT6), leading to FoxO1 deacetylation and decreased PCK1 [26]. Most cancer cells harbor p53 mutations, providing further evidence supporting our finding of low PCK1 expression in CRC cells. We tested PCK1 protein expression in 491 pairs of paraffin-embedded CRC and normal epithelium tissues by IHC and found that almost 90% of CRC expressed lower PCK1 levels than those in non-tumor tissues. We also analyzed associations between PCK1 status and clinicopathological characteristics, to assess which patients were more likely to harbor high PCK1 expression. PCK1 expression was significantly associated with smaller tumor diameter, less bowel wall invasion, and history of alcohol intake. These clinical data suggest that high PCK1 expression may inhibit tumor proliferation. Our functional studies confirmed that PCK1 overexpression could inhibit cell proliferation, while PCK1 deficiency stimulated cell proliferation, in vitro and vivo. Hence, our data confirm that PCK1 acts as an anti-oncogene in CRC. We also demonstrated that patients with low PCK1 expression in stage I–III CRC had significantly decreased DFS; however, PCK1 was not a predictive prognostic biomarker for OS. Since no patients included in this study had received immunotherapy before tumor recurrence or metastasis, the influence on survival of targeted treatment can be ignored. Therefore, our data demonstrate that CRC tumors with low PCK1 expression may be more likely to recur and metastasize.

As a downstream target of the PI3K-AKT signaling pathway, PCK1 can influence the SCAP/SREBP complex to regulate tumor lipid metabolism [6]. PCK1 can also induce tumor cell proliferation by participating in glycolipid conversion [27]. Reactive oxygen species (ROS) generated by lipid peroxidation, oxidative stress, and oxidative DNA damage, are severely toxic to cells, as ROS accumulation can lead to membrane disruption, protein aggregation, and even cell death via autophagy activation [27]. Autophagy is a widespread degradation mechanism in cells which can remove damaged proteins and organelles and maintain cell homeostasis; however, the role of autophagy in tumor cell proliferation has not been fully clarified [20, 28, 29]. In addition, Ma et al. demonstrated that PCK1 knockdown can damage CD8^+^ memory T cell formation and maintenance by decreasing the Glutathione (GSH)/ oxidized glutathione disulfide (GSSG) ratio and increasing ROS levels [23]. This discovery provides a new perspective on the function of PCK1 in autophagy. To verify the role of autophagy in PCK1-overexpressing cells, we used the autophagy inhibitor wort, to inhibit the conversion of LC3B I to LC3B II. The results showed that inhibition of autophagy by wort partially reversed the inhibition effect caused by PCK1 overexpression. Our data indicate that PCK1 inhibits CRC cell proliferation by activating autophagy and confirm that overexpression of PCK1 can inhibit CRC cell proliferation via this mechanism; however, the mechanism underlying PCK1-induced autophagy remains somewhat unclear. As a protein of the ubiquitin—proteasome pathway system, UBAP2L is found in high-density cell fractions containing ubiquitin, and is involved in various tumor-related processes. Further, UBAP2L overexpression is considered a marker of poor prognosis in patients with gastric cancer, glioma, and cervical carcinoma [13–15]. UBAP2L knockdown can also inactivate P38 and suppress CRC cell proliferation via cell cycle arrest and apoptosis [16]. In addition, mechanistic target of rapamycin (mTOR) kinase has essential roles in cell metabolism and proliferation by regulating lysosome biogenesis, as well as glucose and lipid homeostasis [30]. Recent studies have established that mTOR signaling induces protein degradation through autophagy and the ubiquitin-proteasome pathway [31]. PCK1 is downstream of the mTOR pathway [9]; mTOR inhibition up-regulates PCK1 and shuttles glycolytic flux to the gluconeogenesis pathway, thereby inhibiting cellular proliferation in hepatocellular carcinoma and ccRCC [30]. Hence, there is an established relationship between PCK1 and ubiquitin proteasome-induced autophagy. In our study, we identified a novel potential PCK1-mediated autophagy target: Ser 454 phosphorylation of UBAP2L, and our proteomics combined with phosphorylation analysis suggested that PCK1 overexpression can inactivate UBAP2L phosphorylation at Ser 454. Further, we determined that the Ser 454 (A) mutation prevents UBAP2L phosphorylation and reverses PCK1-induced autophagy, while transformation of Ser 454 (A)-mutated UBAP2L reversed PCK1-induced inhibition of CRC cell proliferation. These results suggest that PCK1 activates oncogenic autophagy via down-regulating Ser 454 phosphorylation of UBAP2L, thereby antagonizing CRC growth.

In our study, we found that expression of both PCK1 mRNA and protein was significantly lower in CRC than control tissues, according RNA sequencing, WB, and IHC analyses. In addition, we reveal, for the first time, that PCK1 can inhibit CRC cell proliferation and colony formation by targeting the ubiquitination-autophagy axis (PCK1/UBAP2L Ser 454 phosphorylation/autophagy); this result highlights a molecular mechanism with potential for application in development of new strategies targeting gluconeogenesis-ubiquitination- autophagy pathways.

## Conclusion

In conclusion, we found that PCK1 expression was low in CRC tissues, and that overexpressed PCK1 could inhibit CRC cell proliferation. Mechanistically, PCK1 inactivated UBAP2L phosphorylation at Ser 454 and enhanced autophagy. Our findings identify a novel molecular mechanism involving PCK1 and autophagy, and indicate that PCK1 may be an effective target for CRC treatment strategies.

## Supplementary Information

**Additional file 1.** BAF significantly increased LC3B II expression in PCK1-OE cells relative to that in PCK1-Ctrl and PCK1-sh cells **A–D** The protein expression of LC3B-II/ LC3B-I were significantly increased in cells overexpressing PCK1 relative to that in PCK1-Ctrl after treatment with BAF. **E** and **F** The protein expression of LC3B-II/ LC3B-I remained stable in PCK1-OE cells relative to that in PCK1-Ctrl after treatment with BAF. Actin was used as a control. *P < 0.05, **P < 0.01, and ***P < 0.001.

**Additional file 2.** Rapamycin significantly increased LC3B II expression in PCK1-OE cells. **A** and **B** The protein expression of LC3B-II/ LC3B-I were significantly increased in cells overexpressing PCK1 relative to that in PCK1-Ctrl. *P < 0.05, **P < 0.01, and ***P < 0.001.

**Additional file 3.** Autophagic flux of PCK1-Ctrl SW480 (**A**) or PCK1-Ctrl RKO (**B**) cells after rapamycin or BAF treatment or a combination of both treatments for 4 h. Immunofluorescence dots analysis showed rapamycin could decrease the ratio of GFP-LC3 dots/ RFP-LC3 dots. BAF could iecrease the ratio of GFP-LC3 dots/ RFP-LC3 dots. *P < 0.05, **P < 0.01, and ***P < 0.001.

## Data Availability

The data used to support the findings of this study are available from the corresponding author upon request.
