# Supplementary figures and images for: PCK1 activates oncogenic autophagy via down-regulation Serine phosphorylation of UBAP2L and antagonizes colorectal cancer growth
Source: Cancer Cell Int. 2023 Apr 16;23:68. doi: 10.1186/s12935-023-02894-x (PMC10105959; doi:10.1186/s12935-023-02894-x)

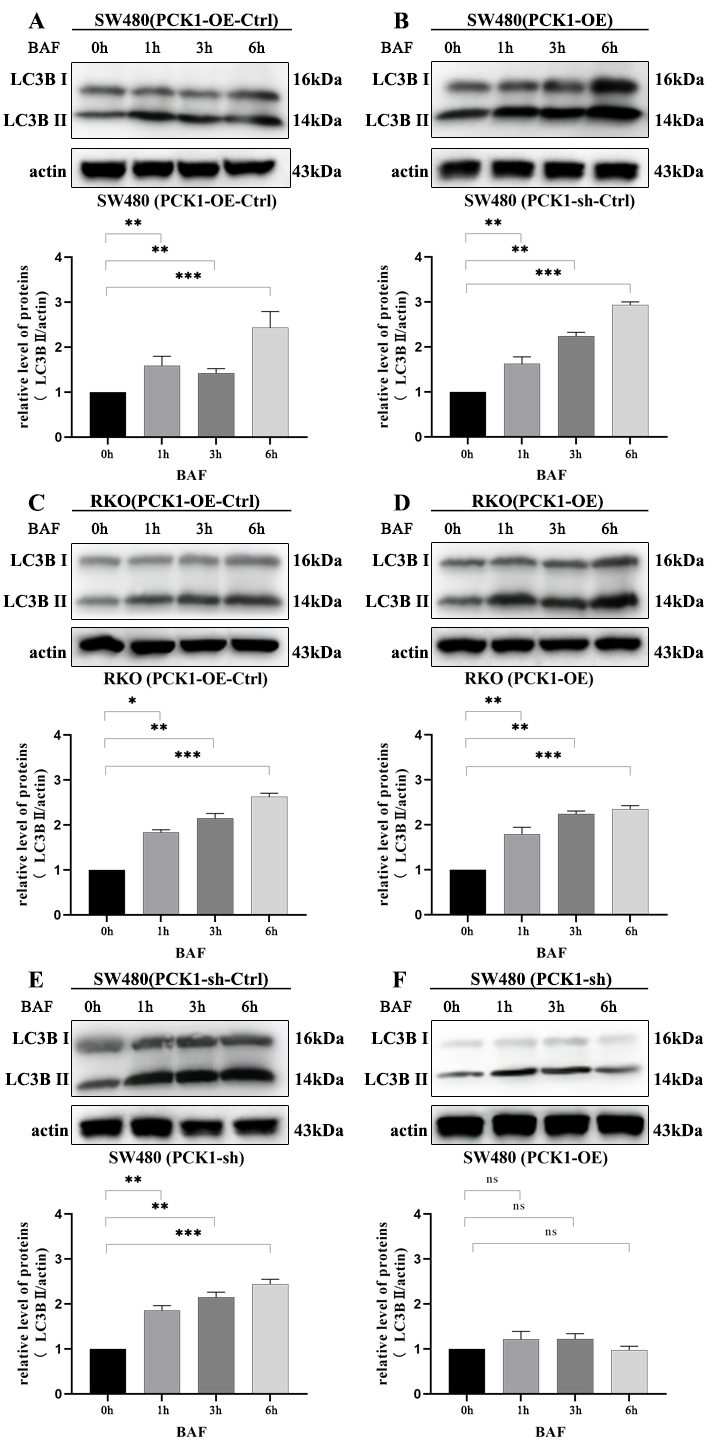

Supplement: Supplementary file 1 — Additional file 1. BAF significantly increased LC3B II expression in PCK1-OE cells relative to that in PCK1-Ctrl and PCK1-sh cells A–D The protein expression of LC3B-II/ LC3B-I were significantly increased in cells overexpressing PCK1 relative to that in PCK1-Ctrl after treatment with BAF. E and F The protein expression of LC3B-II/ LC3B-I remained stable in PCK1-OE cells relative to that in PCK1-Ctrl after treatment with BAF. Actin was used as a control. *P < 0.05, **P < 0.01, and ***P < 0.001. [file 12935_2023_2894_MOESM1_ESM.jpg]

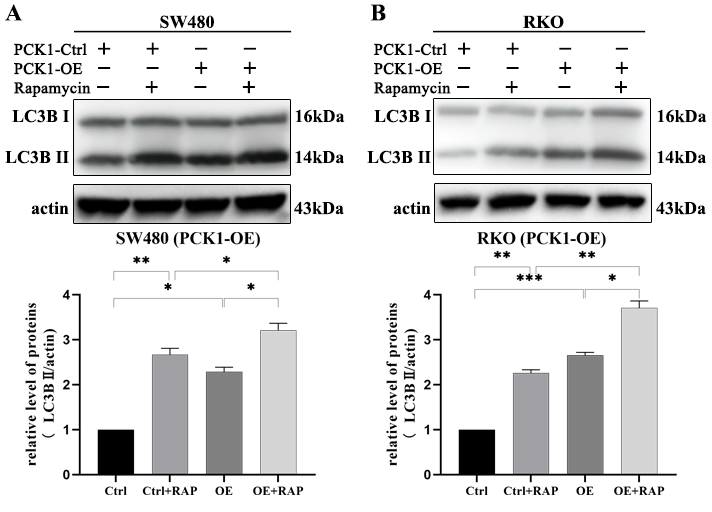

Supplement: Supplementary file 2 — Additional file 2. Rapamycin significantly increased LC3B II expression in PCK1-OE cells. A and B The protein expression of LC3B-II/ LC3B-I were significantly increased in cells overexpressing PCK1 relative to that in PCK1-Ctrl. *P < 0.05, **P < 0.01, and ***P < 0.001. [file 12935_2023_2894_MOESM2_ESM.jpg]

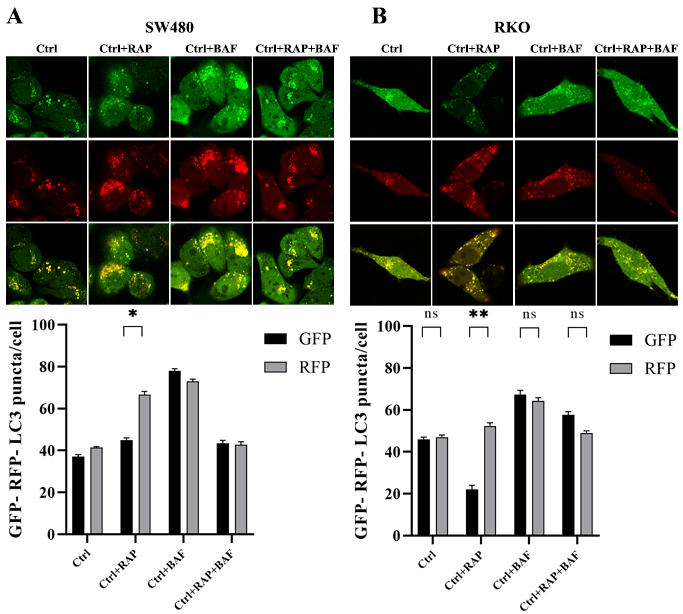

Supplement: Supplementary file 3 — Additional file 3. Autophagic flux of PCK1-Ctrl SW480 (A) or PCK1-Ctrl RKO (B) cells after rapamycin or BAF treatment or a combination of both treatments for 4 h. Immunofluorescence dots analysis showed rapamycin could decrease the ratio of GFP-LC3 dots/ RFP-LC3 dots. BAF could iecrease the ratio of GFP-LC3 dots/ RFP-LC3 dots. *P < 0.05, **P < 0.01, and ***P < 0.001. [file 12935_2023_2894_MOESM3_ESM.jpg]
